# Entropy of balance - some recent results

**DOI:** 10.1186/1743-0003-7-38

**Published:** 2010-07-30

**Authors:** Frank G Borg, Gerd Laxåback

**Affiliations:** 1 University of Jyväskylä, Kokkola University Consortium Chydenius, Health Sciences Unit, Talonpojank. 2B, FIN-67701 Kokkola, Finland

## Abstract

**Background:**

Entropy when applied to biological signals is expected to reflect the state of the biological system. However the physiological interpretation of the entropy is not always straightforward. When should high entropy be interpreted as a healthy sign, and when as marker of deteriorating health? We address this question for the particular case of human standing balance and the Center of Pressure data.

**Methods:**

We have measured and analyzed balance data of 136 participants (young, n = 45; elderly, n = 91) comprising in all 1085 trials, and calculated the Sample Entropy (SampEn) for medio-lateral (M/L) and anterior-posterior (A/P) Center of Pressure (COP) together with the Hurst self-similariy (ss) exponent *α *using Detrended Fluctuation Analysis (DFA). The COP was measured with a force plate in eight 30 seconds trials with eyes closed, eyes open, foam, self-perturbation and nudge conditions.

**Results:**

1) There is a significant difference in SampEn for the A/P-direction between the elderly and the younger groups Old > young. 2) For the elderly we have in general A/P > M/L. 3) For the younger group there was no significant A/P-M/L difference with the exception for the nudge trials where we had the reverse situation, A/P < M/L. 4) For the elderly we have, Eyes Closed > Eyes Open. 5) In case of the Hurst ss-exponent we have for the elderly, M/L > A/P.

**Conclusions:**

These results seem to be require some modifications of the more or less established attention-constraint interpretation of entropy. This holds that higher entropy correlates with a more automatic and a less constrained mode of balance control, and that a higher entropy reflects, in this sense, a more efficient balancing.

## Background

### The attention-constraint interpretation (ACI)

There is a longstanding interest to analyze biological signals in terms of complexity, regularity and chaos. Measures such as entropy, the Hurst ss-exponent and fractal dimensions have become popular. In physiology one can perceive two general lines of interpretations for such measures: (A) One may interpret irregularity and high entropy as signs of a healthy vigilant system; indeed, at the other extreme end we have death which is characterized by a "flat line". Irregularity may thus been seen as a mark of alertness. The system explores the "phase space" and is ready for the unexpected. An impaired system in contrast may become rigid and trapped in repeating patterns unable to successfully cope with new challenges. (B) On the other hand, irregularity and high entropy may be taken as signs that the system is loosing its structure and becoming less sustainable. This is close to the traditional interpretation of entropy as a measure of disorder and noise.

Standing posture is a case in point with regards to these dualistic interpretations. When measuring the excursions during quiet standing in terms of the center of pressure (COP) one may interpret "chaotic" excursions as a sign of poor balance and deficient postural control. On the other hand, chaotic excursions may be also interpreted as a characteristic of a successful vigilant strategy to keep balance. Obviously both interpretations can be correct, but the question is then how to decide which one is the most appropriate one in a case at hand. Or more generally, when is a high entropy, fractal dimension, etc, to be interpreted as a sign of a pathological condition and when as a sign of health [1-4]? This is also intertwined with the issue of complexity vs regularity, and what metric measures which [4]. Roughly speaking entropy is thought to be associated with regularity while various fractal measures are related to complexity, but there is no agreement on this issue. Since there is no unambiguous definition of complexity, theres is no single complexity measure. This motivates the inclusion of a fractal variable in our investigation as a complementary measure, although the interpretation of entropy vis-a-vis balance is the main focus. In the present case we use Sample Entropy [5] as the entropy measure, and the Hurst exponent *α*, based on the detrended fluctuation analysis (DFA) [6], as our fractal measure. The use of DFA in posturographic analysis goes at least as far back as [7] with some more recent investigations such as [8-10].

Table 1 lists a selection of some recent works on the use of entropy in connection with postural control [1,10-18]. Thus a decrease in entropy may be interpreted as sign that more attention is devoted to the balancing which causes a regularization of the COP-curve [13], and conversely that a higher entropy indicates that balancing requires (or gets) less attention [17] and can be handled by the "auto-pilot". While most authors find their hypotheses about entropy confirmed one exception is [10] who finds the larger entropy for elderly to be in conflict with the hypothesis of a decreased complexity with ageing. In our case we also found higher entropy for elderly, which also had higher entropy for the eyes closed condition compared to the eyes open condition, contrasting with [13,17]. The common expectation is to find less *complexity *for the elderly in general [2], which though does not necessarily mean smaller *entropy *[4]. If we adopt the preliminary hypothesis that increasing entropy signifies that lesser attention is devoted to balance control then, in the light of the results for the elderly, it must be modified: Increasing entropy may be interpreted as an *inability *in some circumstances to exert effective attentive control. Thus, an entropy increase during the EC condition could be interpreted as a reduction of an effective attentive control of balance due to the lack of visual input (compensatory proprioceptive inputs are perhaps impaired), the result is therefore a more irregular sway. According to this, ballet dancers have high entropy because they *need not *devote much attention to balance (their well trained "auto-pilot" handles the balancing) while elderly have high entropy because they *cannot *in a similar manner, even if they want to, exert an effective attentive control of balance and "cool down" the system.

**Table 1:** A summary of some studies of entropy in balance

Publication	Study details	Results
[11]	Case study of a 73 y woman with a labyrinthine deficit. Balance training. Dynamic and static tests. Entropy variable: ApEn [28].	Higher entropy after training interpreted as "improved stability", "increased complexity", and as a sign of "a more self-organized system".

[12]	30 young adults. Modified SOT test. Dual task DT (digit recall) vs single task ST. Entropy variable: ApEn.	DT > ST (AP-direction, quiet standing). "Potential of ApEn to detect subtle changes in postural control." Higher ApEn interpreted as a mark of "less system constraint", and a decrease in ApEn as a "change in the allocation of attention."

[13]	30 young adults. QS, EO, EC, DT, ST. DT = uttering words backwards. Entropy variable: SampEn [5] ("regularity") plus scaling exponent, correlation dimension and Ljapunov exponent.	ST: EC < EO; EC: DT > ST. "Regularity of COP trajectories positively related to the amount of attention invested in postural control." Increasing entropy during DT/EC interpreted as an increase in "automaticity" or "efficiency" of postural control.

[14]	10 ballet dancers and 10 track athletes. Foam vs rigid support. Shoulder width stance. Entropy from RQA analysis [35].	Dancers < athletes; EC > EO; foam > rigid. Increasing entropy interpreted as sign of "greater flexibility". Note: the entropy here is calculated differently than SampEn or ApEn.

[10]	14 young and 14 elderly. QS 60 sec and prolonged 30 min. Shoulder width stance (60 sec). Entropy variable: mul-tiscale entropy MSEN [36] plus scaling exponent (DFA [6]).	Old > young (AP-direction); DFA: old < young. Higher entropy for elderly found to be "inconsistent with the hypothesis that complexity in the human physiological system decreases with aging."

[15]	11 low and 11 highly hypnotizable students. 30 sec QS with EC, plus mental computation. "Easy" = stable support; "difficult" = unstable support (foam). Feet position: 2 cm heel-to-heel, 35° splay. Entropy variable: SampEn.	Difficult > easy. "No significant hypnotizability-related modulation was observed."

[16]	10 diabetics II with symptomatic neuropathy, 10 asymptomatic diabetics, and 10 non-diabetics. QS, EO, EC, COP measured in AP-direction. Entropy variable: ApEn.	EC > EO stat. significant only for symptomatic diabetics.

[17]	19 preadolscent dancers and 16 age-matched non-dancers. 20 sec QS with EO, EC, DT. DT = memorize words from audiotape. Entropy variable: SampEn.	Dancers > non-dancers; EC < EO; DT > ST. Higher entropy interpreted as increased "au-tomaticity of postural control."

[18]	19 infants with typical development and 22 infants with delayed development. Sitting postural sway. Entropy variables: symbolic entropy and ApEn.	Delayed < typical in ML-direction. "Healthy postural control is seen to be more complex."

[1]	Case study no. 2, 18 y old collegiate soccer player with cerebral concussion. Entropy variable: ApEn.	Entropy decreased during recovery from concussion. Entropy "can be considered as a measure of system complexity". "Lesser amounts of complexity are associated with both periodic and random states where the system is either too rigid or too unstable."

### COP and the feedback loop

**Figure 1:**
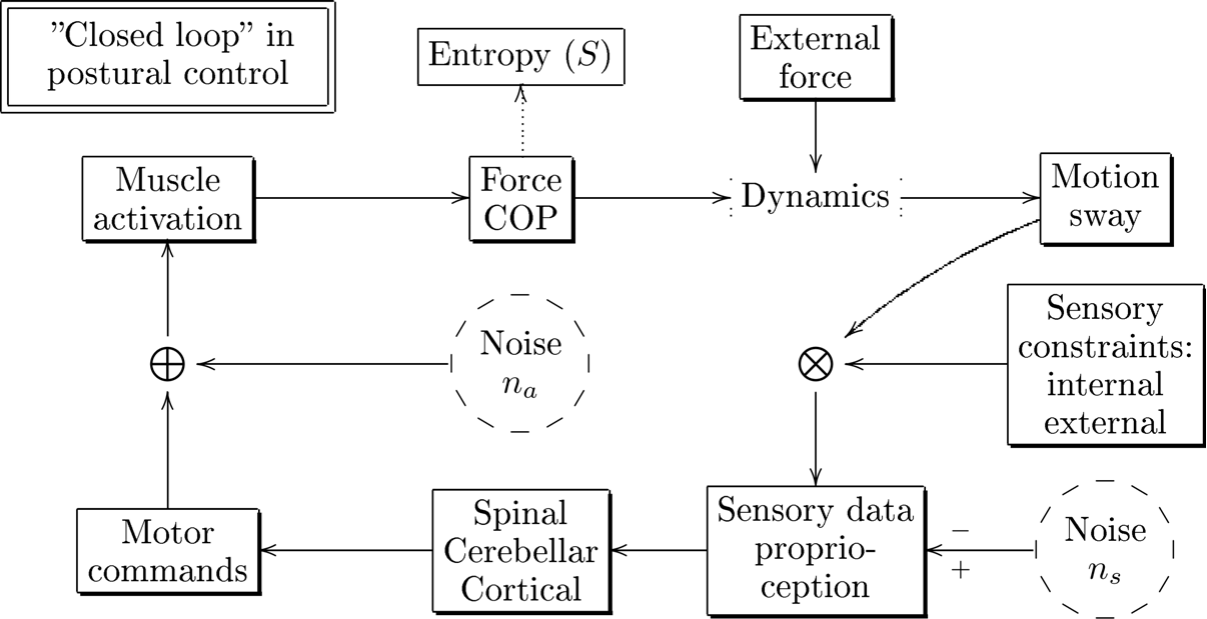
**Balance control system**. A schematic view of the balance control system which describes a closed loop.

At this point it may be a good time to step back a bit and think about what the Center of Pressure (COP) is really measuring. As long as the person stands like an inverted pendulum and controls the posture via ankles, the COP follows closely the Center of Mass (COM) and in this sense gives a good measure of the sway. However, what COP directly measures is the force acting on the force plate via the feet soles. It thus records a sum of the muscular activity of the plantar extensors and flexors, which indeed can be tested with electromyographic (EMG) methods [19]. Therefore a highly variable COP corresponds to a highly variable muscular activity. From a control theory point of view COP is a control variable (the acting force) in a feedback system (see Fig 1), and is *dynamically *closely related to the output variable (sway). This can lead sometimes to confusions when interpreting the results in terms of cause and effect [20]. In Fig 1 noise refers to random or spontaneous processes which in the neural system may be associated with the membrane dynamics. They are depicted as independent sources but they may be under the influence of the feedback loop. Also their output could be placed at alternative points in the diagram. The "+" and "-" signs at the sensory noise arrow emphasize that noise may also have a beneficial effect and *enhance *the sensory threshold e.g. by a process called stochastic resonance [21]. External forces are gravity and perturbations such as a nudge. Given all the acting forces the motion of the system is determined by dynamics (Newtonian mechanics). External sensory constraints include eyes closed condition. Internal constraints may include peripheral neuropathy. The afferent signals are handled principally on three levels. The fastest response is the myotatic stretch reflex (~ 40 ms), then follows the learned automatic responses (~ 100 ms), and finally we have the voluntary responses (> 150 ms). These are annotated as the *spinal*, *cerebellar *and *cortical *components in the diagram.

Strictly speaking the cortical-volitional part breaks the closed loop since the person may decide to change the "setpoints" at any time (with a delay!). In experiments it is though assumed that the participant is instructed e.g. to stand as still as possible and that this constrains his/her responses so as to mimic an automaton (the balance "auto-pilot"). In the diagram we have indicated the output entropy variable(s) *S *calculated from the COP-data. In a closed loop like this the entropy could *prima facie *depend on anything, however if we follow the ACI interpretation we could write the model symbolically as(1)

That is, the basic assumption is that the automatic responses/control increases entropy while the volitional control decreases it. The later effect may be understood as a consequence of the longer volitional response time and consequent more sluggish behaviour. One natural hypothesis then is that volitional control determines the setpoints on a longer time scale, while the automatic control handles the fine tuning toward the setpoints on a shorter time scale. From this interpretation it does not necessarily follow that larger entropy implies smaller COP amplitude. Large entropy may either be associated with a complex fine tuned control (resulting in small COP amplitude) or a an inefficient chaotic control (resulting in a large COP amplitude).

## Methods

### Participants

The group of "elderly" were community dwelling home care clients from a Finnish municipality. They were recruited for a fall risk study. Of these 37 were classified as fallers (F) meaning that they had fallen once or more during the past 12 months at the time of the study. The group of "young" were healthy adults recruited from the same area and were typically office workers. Age and BMI (body mass index) are given in Table 2. All participants gave their written informed consent. The study was approved by the ethical committee.

**Table 2:** Participant characteristics

Group	Number (♂ + ♀)	Age ± SD	BMI ± SD (kg m^-2^)
Elderly Fallers (F)	34 (6 + 28)	81.5 ± 5.7 (68 - 94)	27.3 ± 4.8 (17.7 - 37.6)

Elderly Non-Fallers (NF)	57 (14 + 43)	79.8 ± 6.2 (64 - 91)	29.6 ± 5.3 (20.8 - 46.1)

"Young" (Y)	45 (16 + 29)	38.9 ± 11.6 (17 - 61)	24.3 ± 3.4 (19.5 - 33.8)

### Measurements

The balance measurement was performed using a standard strain gauge force plate (model B4, http://www.hurlabs.com) connected to the PC via USB. The protocol, designed at our lab for fall risk assessment, consisted of the following trials (EO = eyes open; EC = eyes closed):

**EO1 **First EO trial

**EC1 **First EC trial

**EO2 **Second EO trial

**EC2 **Second EC trial

**FOAM **Standing on foam EO (2 cm PE-foam)

**HEAD R **Autohead rotation EO (neutral → left → right → neutral)

**HEAD E **Autohead extension EO (neutral → up → down → neutral)

**NUDGE **Perturbation EO (one forward nudge at the waist level at the beginning of the trial)

Each trial lasted 30 seconds. The foot position (shoes off) was standardized [22]: clearance (heel-to-heel distance) of 2 cm; 30° splay (angle between medial sides of the feet). Arms were held at the sides. A mark on the wall (3 m distance, height 1.5 m) was used for fixing the gaze. The instruction to the participant was to be relaxed (breath normally, etc) and to stand as quiet as possible.

### Analysis

For calculating the Sample Entropy (SampEn) and Detrended Fluctuation Analysis (DFA) we used the computer codes obtained from Physionet [23]http://www.physionet.org/physiotools/. For SampEn we used the "default" parameter values *m *= 2 and *r *= 0.2. Before calculation the COP-data was down sampled from 200 Hz to 10 Hz since: (a) there is little of physiological significance above 10 Hz in the COP signal; (b) it lessens the computational burden of analyzing about 8 hours of data; (c) this down sampling corresponds to a *lag value *also used e.g. by [12]. 10 Hz corresponds to 100 ms which is of the order of the automatic responses and hence also makes physiological sense as a lag time. The sampen function was used with the -n option meaning that the data was normalized before the entropy calculation (mean value is subtracted and the result is then divided by the standard variation). As a measure of the amplitude of COP we have computed its standard deviation denoted *σX *and *σY *for medial-lateral and anterior-posterior direction respectively. For statistical significance level we use *p *< 0.05. For statistical calculations and data visualizations we have used MATHCADhttp://www.ptc.com/products/mathcad/ and the R-package [24]. The two-sample Welch t-test for comparing the means of two sets A and B with unequal variances was calculated by the R-command t.test(A,B). When checking the entropy difference between the EO and EC conditions we have applied the *paired *t-test to *S*(*EO*1) + *S*(*EO*2) and *S*(*EC*1) + *S*(*EC*2). Statistical tests with respects to all trials have been calculated using the averages over the trials for each person. (In R one can use the aggregate command with FUN = mean to obtain the means.)

## Results and Discussion

### Results

**Figure 2:**
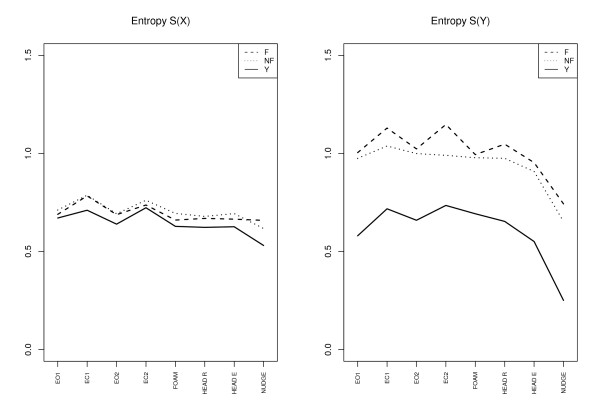
**Entropy**. Entropy for the *X *and *Y *direction for all the trials and the three subgroups: Elderly fallers (F), elderly non-fallers (NF), and young (Y). For each group the value is the group average.

**Figure 3:**
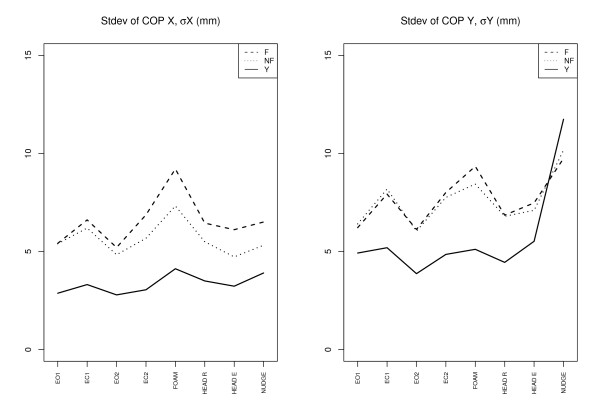
**Center of pressure (COP)**. Standard deviation of COP *X *and COP *Y *for all the trials and the three subgroups: Elderly fallers (F), elderly non-fallers (NF), and young (Y). For each group the value is the group average.

**Figure 4:**
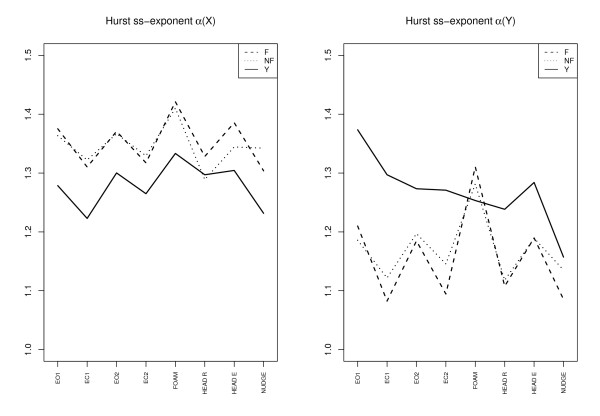
**Hurst exponent**. Hurst ss-exponent for *X *and *Y *direction for all the trials and the three subgroups: Elderly fallers (F), elderly non-fallers (NF), and young (Y). For each group the value is the group average.

The Figures 2, 3 and 4 give an overview of the results. We discuss the notable features for each variable in separate subsections. In the figures we have plotted the mean of the corresponding variable for each subgroup for each trial (F = elderly fallers, NF = elderly non-fallers, Y = "young").

#### Sample entropy

For medial-lateral (X) vs anterior-posterior (Y) a prominent feature is that the groups of elderly have higher entropy for the *Y *-direction: *S*(*Y *) >*S*(*X*) (*p *< 0.0001). A general pattern is the higher entropy in the *X*-direction for eyes closed condition (EC) compared to the eyes open (EO) condition, *S*(*EC, X*) >*S*(*EO, X*) (*p *< 0.0005). For *Y *-direction the elderly *fallers *have a pronounced increase in the eyes closed case compared to the eyes open case (*p *< 0.0001). A final interesting feature is the decrease of *Y *-entropy for the nudge trial for all groups (*p *< 0.0001).

#### COP amplitude

An expected feature is that the "young" in general have a smaller COP amplitude (*p *< 0.0001). One exception is the *Y *-amplitude for the nudge trial. Since the COP *Y *is proportional to the righting torque the relative large COP *Y *for the "young" group in the nudge case reflects the ability to counteract the nudge. The elderly tend to have larger *X *- and *Y *-amplitude with eyes closed compared to eyes open (*p *< 0.0001). The larger *lateral *COP *X *amplitude is a distinguishing feature between the elderly fallers and non-fallers for the foam (*p *= 0.009) and head extension (*p *= 0.04) conditions.

#### Hurst ss-exponent α

We note that mean values *α *for the groups stay well within the range 1 - 1.5 characterizing anti-persistence. For the elderly we have a higher *α*-value in the *X*-direction, *α*(*X*) >*α*(*Y *) (*p *< 0.0001). Another pattern is that *α*(*X*) is lower for the "young" compared with the elderly (*p *< 0.0002). A conspicuous feature for the elderly is that α goes up and down from trial to trial. This is true also for the "young" in the *X*-direction but not so in the *Y *-direction.

#### Relations

**Figure 5:**
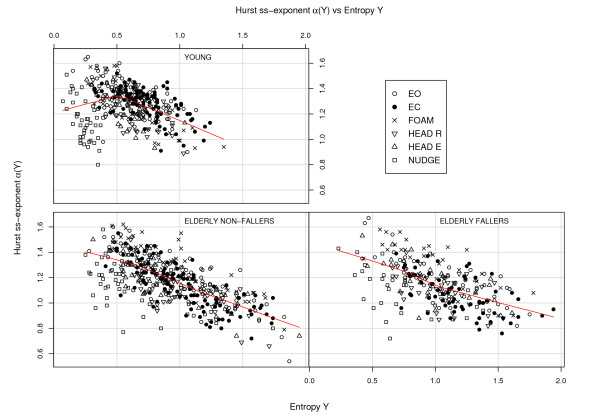
**Hurst exponent vs entropy**. Hurst ss-exponent *α*(*Y *) vs entropy *S*(*Y *) for all the trials and the three subgroups. The lines show the local polynomial regression fit "loess" (W S Cleveland) which can be produced by the R-function panel.smooth.

**Figure 6:**
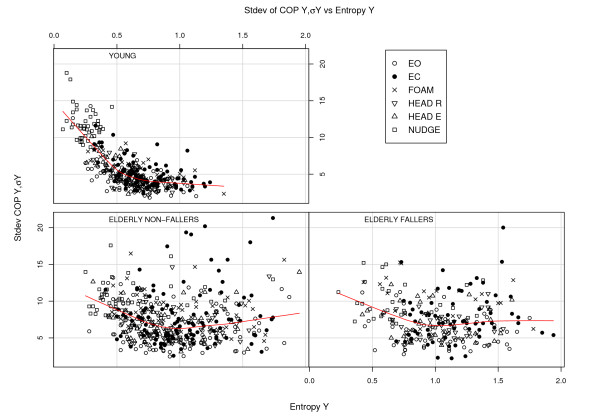
**Entropy vs COP**. Entropy *S*(*Y *) versus standard deviation *σ*(*Y *) of COP *Y *for all the trials and the three subgroups. The lines show the local polynomial regression fit "loess".

For all the variables we have a positive correlation between the *X*- and *Y *-components. What is more interesting are the negative correlations for the pairs Entropy *X*, Hurst *α*(*X*) (corr. = -0.68, *p *< 0.0001) and Entropy *Y *, Hurst *α*(*Y *) (corr. = -0.84, *p *< 0.0001), see Fig 5. A negative correlation is expected as far as a higher *α *value is associated with a smoother signal which in general implies a smaller entropy. The nudge tests deviate a bit from the general pattern; this was the condition where entropy took a plunge. Of interest is also the question whether there is some relation between entropy and COP amplitude. Fig 6 depicts entropy *S*(*Y *) for the Y-direction plotted against the COP *Y *amplitude *σY *. For the "young" there is a quite distinct pattern with a "knee" around *S*(*Y *) = 0.5 as in Fig 5. Discounting the nudge trials then only the "young" group has a significant correlation between entropy *S*(*Y *) and Y-amplitude *σY *(-0.39, *p *< 0.008).

## Discussion

The attention-constraint interpretation (ACI) seems to be in accord with lowering of entropy *S*(*Y *) in the nudge trial (Fig. 2). However, the higher entropy in the eyes closed case, *S*(*EC*) >*S*(*EO*), seems, *prima facie*, to be at variance with the ACI and some results in the literature, see e.g. [13,17] or Table 1. We may though understand the higher entropy in EC case, despite an "increasing cognitive involvement in postural control" [[13], p. 1], if the lack of visual cues cannot be compensated for by other proprioceptive cues. That is, lack of sensory information through sensory deprivation, or impairment, may imply that an increase of cognitive involvement does not translate into a corresponding constrained mode of balance. The pilot is so to speak flying blinded. Suppose the attentive control works by increasing the deterministic component in relation to the noise and that it may in this way lead to decreased entropy. However, if the sensory input is affected by noise then the output of the deterministic control will also be accordingly affected by noise, and we may see an increase in entropy instead of a reduction. The higher entropy *S*(*Y *) for the elderly may be interpreted along these lines as an effect of a more impaired (noisy) sensory system which provides less precise input for the balance control. This is supported also by Fig. 6 where the data for elderly show an increase in the scatter of COP *Y *when entropy is above about 1 unit. For the young, however, an increased entropy *S*(*Y *) is associated with a smaller COP *Y *. In this case increased entropy apparently signifies a more fine tuned control and not so much the contribution from noise.

One finding related to fallers vs non-fallers was the greater medial-lateral (M/L) sway for fallers during the foam and head rotation conditions. M/L-sway (foam) *σX *≥ 10 mm indicates for the elderly roughly an odds ratio of 4.5 for belonging to the fallers group. Several other studies have also implicated increased lateral sway as a marker for fall risk, see e.g. [25,26]. A novel feature here may be the increased SampEn for the *anterior-posterior *COP *Y *during eyes closed condition (EC) for the elderly fallers relative to the non-fallers. This suggests that one should make further studies of the usefulness of this entropy variable as a fall risk indicator. The reason why a similar entropy increase does not show up for the M/L-sway for the EC condition is a bit of a mystery, but maybe is related to the somewhat different control mode (shifting the weight between the legs) of the M/L-sway for bipedal quiet standing, compared to the control of the A/P-sway.

If we wish to establish a canon of entropy interpretation, we could proceed by measuring entropy vs COP for various groups and conditions, as exemplified by Fig. 6. Those groups which are known to have excellent balance would then define the optimal entropy relation. Hopefully this could then be followed up by a convincing theoretical framework. With an appropriate test protocol one could draw an entropy-COP diagram for an individual that could yield further clinically useful information on the weak/strong points of the balance control. A complementary approach would be to use brain imaging techniques during balancing tasks [27] to reveal whether some specific functional areas, if such areas can be identified, are correlated with the entropic measures.

## Conclusions

The data presented here provide further evidence that entropy is a variable that may complement the traditional posturographical variables. Comparison of results from young and elderly reveals though that more work is needed to identify the correct physiological interpretation of entropy in a given situation. One way to proceed is to measure the entropy-COP relation for various groups of people and conditions. Those known to have excellent balance control would define the optimal entropy relation. Of clinical importance is to find those conditions (test protocols) that yield a maximum of information about deficiencies of the balance control, yet are safe and simple to administer.

## List of abbreviations

*α*: Hurst self-similarity (ss) exponent; ACI: attention-constraint interpretation; ApEn: approximate entropy; A/P: anterior-posterior; BMI: body mass index; COP X: medio/lateral center of pressure; COP Y: anterior/posterior center of pressure; DFA: detrended fluctuation analysis; EC: eyes closed; EO: eyes open; H: Hurst parameter; S: (sample) entropy; M/L: medial-lateral; *σ*: standard deviation.

## Competing interests

All authors acknowledge that we do not have any financial or personal relationships with other people or organizations that would inappropriately influence the results of this study.

## Authors' contributions

FB has analyzed the data and prepared the manuscript. GL has collected the data, and has also contributed to the design of the tests. All authors have read and approved the final manuscript.

## Appendix

### Entropy

Approximate Entropy (ApEn [28]) and Sample Entropy (SampEn [5]) which are commonly used in physiological applications belong to the dynamic category. Dynamic entropy is concerned with the predictability of the signal. If we know the signal up to time *t*_0_, how well can we predict its succession for times *t *>*t*_0_? In terms of information the question be formulated as follows: If we know the signal for a time interval [*t*_*i*_, *t*_*i*+1_] how much additional information is needed to predict the signal for the time interval [*t*_*i*+1_, *t*_*i*+2_]? For a simple deterministic signal no new information is needed once we know the "formula" which generates it. On the other extreme, for a completely random signal we need to know the whole signal in advance in order to "predict" it. We can also formulate the information excess as the entropy produced per time of evolution, a concept which was advanced by Kolmogorov (1958) and Sinai (1959) (Kolmogorov-Sinai entropy, KS, [[29], p. 193]). ApEn and SampEn are simplified numerical estimates of the KS-entropy. Generally speaking these entropies approximate the expression ln(1/*P*), where *P *is the conditional probability that if two sets *z*_*i*_, *z*_*j *_of *m *consecutive data points (*d *is the *lag*, typically taken as *d *= 1 depending on the sampling rate),(2)

are close to each other, ||*z*_*i *_- *z*_*j *_||<*r *· *SD*, then so will the next following points be too, |*x*_*i*+*md *_- *x*_*j*+*md*_| Therefore ApEn and SampEn can be seen to estimate the degree of "surprise" in the data. Here the distance ||*z*_*i *_- *z*_*j *_|| between two sequences is defined as the largest absolute difference between any two pairs of data points from the sequences. The distance is measured in terms of the fraction *r *of the standard deviation *SD *of the time series. Typical choices for the parameters are *m *= 2 which is the so called *embedding dimension*, and *r *= 0.2 for the so called *tolerance*; for more elaborate methods of selections of these parameters see [30,31]. In our case *m *is restricted by the size of the downsampled time series (300 points). As a rule thumb one needs about 10^*m *^- 20^*m *^data points [32].

### The Hurst self-similarity (ss) exponent α

The Hurst parameter *H *(after the hydrologist Harold Hurst) is related to a scaling property of time series *x*(*t*) and is also though of as one of the metrics for *complexity *(for which there is no universal definition [33]). The idea is that if we appropriately rescale the time axis and the ordinate then the curve "looks similar". One mathematical rendering of this idea is that the mean variance (*x*(*t *+ Δ) - *x*(*t*))^2 ^depends on Δ as a power Δ^2*H*^,(3)

One example is a type of random motion called Brownian motion for which *H *= 0.5. Basically we could determine *H *from numerical data by computing the variance (3) for series of values Δ and map variance against Δ using logarithmic axes. The detrended fluctuation analysis (DFA) [6] is a variant of this method which is applied to the *cumulative sum y *of *x*, *yk *= ∑_*i*≤*k *_*x*_*i*_, instead of *x *itself. This is for numerical robustness reasons. Secondly the data is divided into blocks of sizes *n*, and for every block the data is approximated by a linear function *yn *by which we obtain the "detrended data" *y *-*yn*. Finally the "variance" is computed ∑_1≤*k*≤*N *_(*yk *-*yn*_*k*_)^2^/*N *as the mean square the detrended data. If this depends on *n *as *n*^2*α *^then *α *is defined as the *self-similarity parameter *of *x*. For time series which satisfy the self-similarity property we have the theoretical relation *α *= *H *+ 1. Because α is based on the cumulative sum *y *it covers a bigger range 0.5 <*α *< 2 than *H *which is restricted to the range 0 <*H *< 1. An important property is that signals *x *with 0 <*H *< 0.5 (1 <*α *< 1.5) exhibit so called *anti-persistence *meaning that subsequent increments in *x *tend to anti-correlate,

For 0.5 <*H *< 1 (1.5 <*α *< 2) we have the opposite property called *persistence*. For a pendulum, as an example, we may expect persistence for small time intervals since it tends to continue its motion in the same direction. For longer time intervals we expect anti-persistence since the pendulum swings back. A smaller *α*-value for quiet standing COP can thus be interpreted as a higher degree of anti-persistence; that is, a higher proportion of rapid corrective impulses.

For a self-similar curve the power spectrum *P*_*x*_(*f*), as a function of the frequency *f*, has the form(4)

This relation suggests that with increasing *α *(or *H*) the curve becomes increasingly smooth since the higher frequency components are suppressed. Finally, in the case of self-similar time series *x*(*t*), the Hurst ss-exponent can be related to the fractal dimension *D *of the graph (*t*, *x*(*t*)) as *D *= 3 - *α *= 2 - *H *[[34], p. 60].
